# The High Prevalence of Testicular Adrenal Rest Tumors in Adult Men With Congenital Adrenal Hyperplasia Is Correlated With ACTH Levels

**DOI:** 10.3389/fendo.2019.00335

**Published:** 2019-06-04

**Authors:** Rossella Mazzilli, Antonio Stigliano, Michele Delfino, Soraya Olana, Virginia Zamponi, Cristina Iorio, Giuseppe Defeudis, Danilo Cimadomo, Vincenzo Toscano, Fernando Mazzilli

**Affiliations:** ^1^Andrology Unit, Department of Clinical and Molecular Medicine, Sant'Andrea Hospital, University of Rome “Sapienza”, Rome, Italy; ^2^Endocrinology Unit, Department of Clinical and Molecular Medicine, Sant'Andrea Hospital, University of Rome “Sapienza”, Rome, Italy; ^3^Unit of Endocrinology and Diabetes, Department of Medicine, Campus Bio-Medico University of Rome, Rome, Italy; ^4^G.E.N.E.R.A Centers for Reproductive Medicine, Rome, Italy

**Keywords:** congenital adrenal hyperplasia, testicular lesion, semen analysis, male infertility, testicular adrenal rest tumor, azoospermia, testosterone, cortisol

## Abstract

**Introduction:** The aims of this study were to determine the prevalence of testicular-adrenal rest tumors (T-ARTs) in patients with congenital adrenal hyperplasia (CAH) and to evaluate the related ultrasound (US) features, hormonal profiles, and semen parameters. Therefore, we attempted to understand the potential impact of adrenocorticotropic hormone (ACTH) on the persistence or disappearance of T-ART.

**Methods:** We conducted a longitudinal cohort study including patients with CAH who were undergoing treatment with cortisone and, when indicated, fludrocortisone replacement therapy. We performed andrological examinations, US of the testis, hormone profiling, and semen analysis.

**Results:** Of the 25 patients (mean ± SD age, 32.2 ± 7.5 years), T-ARTs were detected by US in 14 (56.0%) patients. The mean ± SD diameter of the lesions was 13.2 ± 6.8 mm. Among 3 (21.4%) patients, the lesions were observed in one testis, whereas both testes were affected in the remaining 11 (78.6%) patients. The lesions were hypoechoic in 12 (85.7%) patients and hyperechoic in 2 (14.3%). Plasma ACTH and 17-hydroxyprogesterone (17-OHP) levels were significantly higher in patients with T-ART than in patients without lesions (319.4 ± 307.0 pg/ml and 12.4 ± 2.7 ng/ml vs. 33.5 ± 10.7 pg/ml and 8.2 ± 1.8 ng/ml, respectively; *p* < 0.01). The mean values of sperm concentration and motility were significantly lower in patients with T-ART than in patients without lesions (12.1 ± 12.4 × 10^6^ cells/ml and 18.4 ± 11.1% vs. 41.5 ± 23.2 × 10^6^ cells/ml and 30.8 ± 15.4%, respectively; *p* < 0.05). Logistic regression analysis showed ACTH level as a significant predictor of T-ART (*p* < 0.05). In patients with T-ART, the dose of hydrocortisone was increased by ~25–30%, while the fludrocortisone treatment remained unchanged. After 6 months of steroid treatment, patients underwent US and hormonal evaluation. Disappearance and a reduction in T-ART were observed in 6 (42.9%) and 5 (35.7%) patients, respectively; a reduction in ACTH levels (from 319.4 ± 307.0 to 48.1 ± 5.1 pg/ml; *p* < 0.01) was reported. A significant correlation between ACTH level reduction and T-ART diameter reduction was observed (*p* < 0.5; *r* = 0.55).

**Conclusions:** T-ARTs were detected in 56% of patients with CAH and were associated with impaired semen parameters. However, these lesions are potentially reversible, as demonstrated by the disappearance/reduction after adjustment of cortisone therapy and by the reduction in plasma ACTH level. Our study supports the importance of periodic US evaluation and maintenance of plasma ACTH levels within the normal range in men with CAH.

## Introduction

Congenital adrenal hyperplasia (CAH) is a group of rare inherited autosomal recessive disorders that occurs at an incidence of 1/10,000–20,000 (1, 2). In ≥90% of cases, CAH is caused by a mutation in the CYP21A2 gene that leads to a deficiency of 21-hydroxylase (CYP21), due to CYP21A2 gene mutations.

This enzyme converts 17-hydroxyprogesterone (17-OHP) into 11-deoxycortisol and progesterone into 11-deoxycorticosterone—the precursors of cortisol and aldosterone, respectively. Consequently, the levels of cortisol and, in some cases, aldosterone reduce.

Testicular adrenal rest tumors (T-ARTs) represent islands of adrenal tissue that remain within the gonads during embryonic development. The presence of these lesions has been described in patients with CAH, with a prevalence ranging from 0 to 94% (3–10).

Adrenal rest tissue may migrate with the gonads during their descent (4). Furthermore, the deficit in cortisol production in CAH causes a reduction in negative pituitary feedback, resulting in an increase in adrenocorticotrophic hormone (ACTH) levels. This causes adrenal gland hypertrophy and the overstimulation of ectopic adrenal cells located in the testes. T-ARTs can cause seminal alterations owing to the effects of compression exerted on the rete testis. Moreover, high levels of ACTH indirectly interfere with spermatogenesis in these patients. In fact, increased levels of ACTH induce an adrenal-derived androgens excess that can cause negative feedback on the pituitary and hypothalamus, with a consequent suppression of gonadotropins and testicular testosterone production. Therefore, this mechanism can impair spermatogenesis (3).

In particular, in cases of the heterozygous form of the disease, T-ARTs may remain unrecognized for several years. Previous studies have confirmed the high prevalence of T-ARTs in patients with CAH, and it was hypothesized that ACTH plasma levels play a role in the pathogenesis (8).

In CAH patients, the diagnosis of oligozoospermia or azoospermia, or the detection of one or more small size masses in the testis may lead to the suspicion of T-ART. Furthermore, the presence of a testicular mass could be interpreted as a malignancy.

The aims of this study were to determine the prevalence of T-ART in patients with CAH and to evaluate the ultrasound (US) features, hormonal profiles, and semen parameters. Therefore, we attempted to understand the potential impact of ACTH on the persistence or disappearance of T-ART.

## Materials and Methods

Male patients with CAH, who were referred to our endocrinological unit between January 2006 and September 2017, were included in this longitudinal cohort study and underwent an andrological evaluation to evaluate possible complication of CAH. Inclusion criteria were male sex and CAH due to 21-hydroxylase deficiency. Exclusion criteria were the presence of CAH due to other enzymatic defects in cortisol synthesis.

The patients had been receiving glucocorticoids (hydrocortisone 15–25 mg/m^2^, thrice daily) and, when indicated, fludrocortisone (0.05–0.2 mg, 1–2 times/daily) replacement therapy for ≥1 year.

Written informed consent was obtained from all individual participants who were included in the study. The study was conducted according to “Sapienza” University of Rome Ethics Committee.

The study included andrological clinical examinations, US of the testis, hormonal profiling, and standard semen analysis. The andrological examination was aimed at evaluating the testis (shape, size, and appearance), epididymis, penis, and body hair distribution, and the possible presence of gynecomastia. US and color Doppler sonography were performed with a linear transducer probe of 7 MHz (Philips Ultrasound Machine HDI 4000). To study the morphology, testicular size was evaluated using an ellipsoid formula (length × width × depth × 0.52) (11). Blood samples were obtained at 8:00 am; the plasma levels of luteinizing hormone (LH), follicle stimulating hormone (FSH), testosterone, ACTH, 17-OHP, and cortisol were measured. Chemiluminescence microparticle immunoassay (CMIA) was used to detect testosterone; chemiluminescence immunoassay (CLIA) for 17-OHP, FSH, and LH; and LIAISON CLIA for ACTH and cortisol. Serum potassium and sodium levels were analyzed by ion-selective electrode method, fasting blood glucose was analyzed by enzymatic methods, and insulin was analyzed by microparticle enzyme immunoassay. Semen analysis was carried out according to 1999–2010 WHO guidelines (12, 13). Samples of sperm were collected by masturbation after sexual abstinence from 2 to 7 days. Sperm concentration and motility were evaluated using the Superimposed Image Analysis System (SIAS) as described previously (14).

### Statistical Analysis

Continuous data were described as absolute values, mean ± standard deviation (SD), and range. Categorical data were described as absolute number, percentage frequency, and 95% confidence intervals (CI). We used Fisher's exact test for analyzing categorical data; *p* < 0.05 was considered statistically significant. The software R version 2.14.2 (Free Software Foundation, Inc., USA) was used for statistics and logistic regression analyses.

## Results

All patients were post-pubertal and the mean ± SD of the age was 32.2 ± 7.5 years (range, 18–43 years). The mean ± SD of hydrocortisone dosage was 18.5 ± 2.6 mg/day (range, 15–22.5 mg/day), and all patients were in normal weight range (BMI mean ± SD: 22.5 ± 1.1 kg/m^2^; range, 20.8–24.1 kg/m^2^). Only 2/25 men had previously conceived.

The physical examination of the scrotum showed a palpable testicular mass only in one patient (4.0%). The remaining patients did not have any masses. None of the patients showed objective anomalies of testis, epididymis, penis, and body hair distribution or gynecomastia.

The testicular size, evaluated using US, was within the normal range in all patients (mean ± SD in right testis: 17.7 ± 1.9 ml; range, 15–21.3 ml; mean ± SD in left testis: 17.2 ± 1.7 ml; range, 14.9–20.7 ml).

T-ARTs were detected by US in 14/25 (56.0%) patients. The mean diameter of the lesions was 13.2 ± 6.8 mm (range, 4–26 mm). In 3/14 patients (21.4%), the lesion involved only one testis, while in 11/14 patients (78.6%), both the testes were affected.

In 12/14 patients (85.7%), the lesions were largely hypoechoic. In the remaining 2/14 patients (14.3%), the lesions were largely hyperechoic. Color Doppler US showed an absence of flow in 10/14 (71.4%) patients, perilesional flow in 3/14 (21.4%) patients, and perilesional and intralesional flow in the remaining patient (1/14; 7.1%).

No differences in testicular size and in potassium, sodium, insulin, and fasting blood glucose concentration were observed in patients with and without T-ART. Plasma levels (mean ± SD values) of ACTH, 17-OHP, FSH, LH, and testosterone are shown in Table 1. The mean value of ACTH was above the normal range in all patients with T-ART. Both ACTH and 17-OHP levels were significantly higher in patients with T-ART (319.4 ± 307.0 pg/ml and 12.4 ± 2.7 ng/ml, respectively) than in patients without lesions (33.5 ± 10.7 pg/ml and 8.2 ± 1.8 ng/ml, respectively; *p* < 0.01).

**Table 1:** Hormonal profile in patients without testicular adrenal rest tumor and with testicular adrenal rest tumor at basal condition and after 6 months of modified glucocorticoid treatment.

	**No T-ART (*n* = 11) Mean ± SD (min–max)**	**T-ART basal (*n* = 14) Mean ± SD (min–max)**	**T-ART 6 months (*n* = 14) Mean ± SD (min–max)**	***P*-value**
ACTH (pg/ml)	33.5 ± 10.7 (11–45)	319.4 ± 307.0 (55–950)	48.1 ± 5.1 (39–55)	<0.01
17-OHP (ng/ml)	8.2 ± 1.8 (5.1–10.2)	12.4 ± 2.7 (8.9–18.4)	8.0 ± 1.2 (6.3–10.3)	<0.01
FSH (mIU/ml)	3.6 ± 1.7 (1.2–6.4)	2.8 ± 1.4 (1.1–6.0)	3.0 ± 1.4 (1.6–6.1)	N.S.
LH (mIU/ml)	2.9 ± 1.1 (1.8–5.1)	2.6 ± 1.3 (1.2–4.9)	2.6 ± 1.2 (1.3–5.1)	N.S.
Testosterone (ng/ml)	4.5 ± 0.9 (3.1–6.1)	5.3 ± 1.3 (3.1–8.0)	5.2 ± 1.3 (3.4–8.1)	N.S.

*T-ART, testicular adrenal rest tumor; T-ART basal, basal condition; T-ART 6 months, hormonal profile after 6 months*.

Semen analysis showed azoospermia in 2 (14.3%) patients and normospermia in 3 (21.4%) patients with T-ART. The mean values of semen volume, sperm concentration, and progressive motility were significantly lower in patients with T-ART (2.7 ± 0.7 ml, 12.1 ± 12.4 × 10^6^ cells/ml, and 18.4 ± 11.1%, respectively) than in patients without lesions (1.9 ± 0.5 ml, 41.5 ± 23.2 × 10^6^ cells/ml, and 30.8 ± 15.4%, respectively; *p* < 0.05) (Table 2). None of the patients with T-ART had previously conceived.

**Table 2:** Semen parameters in patients without and with testicular adrenal rest tumor.

	**No T-ART (*n* = 11) Mean ± SD (min–max)**	**T-ART (*n* = 14) Mean ± SD (min–max)**	***P*-value**
Volume (ml) (>1.5 ml)	2.7 ± 0.7 (1.6–4.1)	1.9 ± 0.5 (1.1–3.0)	<0.05
N/ml (>15 × 10^6^)	41.5 ± 23.2 (4.0–84.0)	12.1 ± 12.4 (0–36.0)	<0.01
Progressive motility (%) (>32%)	30.8 ± 15.4 (3.0–50.0)	18.4 ± 11.1 (0–38.0)	<0.05
Atypical form (%) (<96%)	69.8 ± 8.5 (61.0–89.0)	76.7 ± 8.3 (59.0–96.0)	N.S.

*T-ART, testicular adrenal rest tumor*.

Logistic regression analyses were conducted to investigate all the possible basal confounders upon the presence of T-ART (i.e., age, testis size, BMI, hydrocortisone dosage, basal ACTH, LH, FSH, testosterone, and 17-OHP) and to evaluate possible correlation between T-ART size and hormonal level and between T-ART size and semen parameters. Only the ACTH level (*p* < 0.05) was shown as a significant predictor of T-ART.

In patients with T-ART, the dose of hydrocortisone was increased by ~25–30% with respect to the body mass surface of the patients (from 18.0 ± 2.2 mg/day; range, 15–22.5 to 22.5 ± 2.2 mg/day; range, 17.5–25 mg/day), while the fludrocortisone treatment remained unchanged. After 6 months of steroid treatment, all patients underwent US and hormonal evaluation, and 10/14 patients also underwent semen analysis (Tables 1, 3).

**Table 3:** Semen parameters in patients with testicular adrenal rest tumor who repeated semen analysis after 6 months of modified glucocorticoid treatment.

	**T-ART basal (*n* = 10) Mean ± SD (min–max)**	**T-ART 6 months (*n* = 10) Mean ± SD (min–max)**	***P*-value**
Volume (ml) (>1.5 ml)	1.8 ± 0.4 (1.1–2.2)	1.9 ± 0.3 (1.4–2.3)	N.S.
N/ml (>15 × 10^6^)	8.7 ± 8.8 (0–29)	15.4 ± 12.7 (0–41)	N.S.
Progressive motility (%) (>32%)	16.9 ± 6.7 (10.0–30.0)	16.8 ± 8.0 (0–28.0)	N.S.
Atypical form (%) (< 96%)	73.8 ± 6.0 (63.0–83.0)	75.6 ± 0.4 (61.0–96.0)	N.S.

*T-ART, testicular adrenal rest tumor; T-ART basal, basal condition; T-ART 6 months, semen parameters after 6 months*.

**Figure 1:**
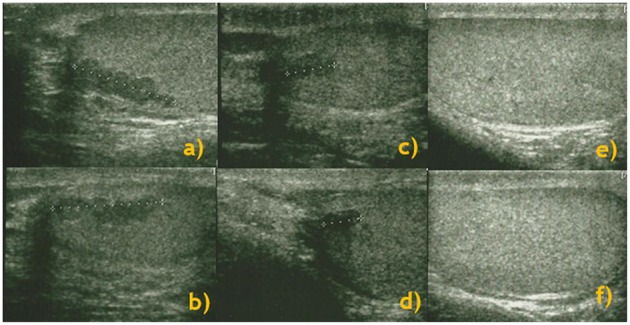
Longitudinal study: testis ultrasound performed at baseline [right **(a)** and left **(b)** testes], and 3 months [right **(c)** and left **(d)** testes] and 6 months [right **(e)** and left **(f)** testes] after cortisone therapy and a consequent improvement in ACTH levels.

US showed a disappearance or reduction in 11 (78.6%) T-ART patients. In particular, in 5 (45.5%) patients, we observed a reduction in the lesion diameter, while in the remaining 6 (54.5%) patients, there was a total disappearance of lesions. Figure 1 represents the evolution of lesions after 3 and 6 months of modified glucocorticoid treatment.

A significant reduction in plasma ACTH and 17-OHP levels was observed (from 319.4 ± 307.0 to 48.1 ± 5.1 pg/ml and from 12.4 ± 2.7 to 8.0 ± 1.2 ng/ml, respectively; *p* < 0.01), while the other hormonal levels remained similar to the basal condition (Table 1). A linear regression analysis and a Pearson correlation test highlighted a correlation between ACTH level reduction and T-ART diameter reduction (*p* < 0.5; *r* = 0.55).

The follow-up of semen parameters was obtained in 10/14 patients with T-ART. An improvement in sperm number (from 8.7 ± 8.8 × 10^6^ to 15.4 ± 12.7 × 10^6^ cells/ml) was observed in such patients, but the comparison was not statistically significant (Table 3). Probably, this is due to the small number of patients. The other semen parameters remained unchanged.

Finally, none of these patients showed pathologically high blood glucose values or insulin resistance.

## Discussion

CAH represents a group of autosomal recessive disorders characterized by enzymatic defects in the cortisol biosynthesis pathway. Treatment of CAH includes the use of glucocorticoids and, when indicated, mineralocorticoid replacement therapy. Clinical manifestations in the neonatal stage include adrenal insufficiency symptoms (e.g., vomiting, dehydration), ambiguous genitalia, and skin hyperpigmentation (2). A complication in adult patients with CAH is the presence of intratesticular area of ectopic adrenal tissue in the testis. The prevalence of this condition among men affected by CYP21 deficiency varies between 0 and 94% (3–10). The variability could be attributed to heterogeneity in the selection of patients, age, and the various glucocorticoid treatment regimens. In fact, Kim et al. (15) suggested that the prevalence of T-ART is high in young men with CAH, but low in infants or elderly patients.

Here, we studied the prevalence of T-ART, the hormonal profile, the US features, and the semen parameters in patients with CAH. According to previous studies (8, 16), the prevalence of T-ART could be higher than 50%. US features showed a higher prevalence of bilateral and hypoechoic area and the absence of intra-lesional flow in the T-ART. Differential diagnosis of other testicular lesions, such as Leydig cell tumors, could be performed following certain criteria that suggest T-ARTs are bilateral in a high percentage of cases; patients with Leydig cell tumors had gynecomastia. The definitive histological differential diagnosis is based on the absence of Insulin-like 3 and Reinke crystalloids in T-ART (17).

Ahmad et al. suggested the use of MRI in a patient with CAH (18). Adrenal cortex is isointense with muscle on T1- and T2-weighted images. T-ARTs have the same characteristics as the testicular parenchyma. Injection of gadolinium revealed a homogeneous enhancement. To date, MRI is not considered a gold standard in the study of the testes. In the present study, more than 80% of the patients had bilateral lesions, and none of the patients showed gynecomastia.

Importantly, the study showed a reduction or a disappearance of the lesions in more than 75% of the cases after a modification of the cortisone therapy and a significant reduction in plasma ACTH level. The result was further corroborated by (a) the detection of high ACTH levels in all patients with T-ART and normal levels in patients without testicular lesions, (b) logistic regression analysis showing ACTH level as a significant predictor of T-ART, and (c) the significant correlation between reduction of ACTH levels and reduction of T-ART diameters. The role of ACTH in the induction of T-ART has been previously shown also in patients with Cushing syndrome who developed a testicular mass ≥10 years after bilateral adrenalectomy (19).

For this reason, T-ART should be suspected not only in CAH patients, but in all men with potentially high ACTH levels (i.e., Cushing syndrome, Addison disease, and adrenalectomy).

Treatment with excessively high dose of glucocorticoids must be avoided as it may lead to the development of diabetes and obesity, insulin resistance, and osteoporosis (1, 2, 20, 21). Glucocorticoid-induced hyperglycemia depends on the total glucocorticoid dose and duration of therapy and may occur also in <1 month (22). Therefore, metabolic parameters should be examined. In the present study, none of these patients developed diabetes or insulin resistance during follow-up.

Our results also underlined, in accordance with previous studies (10, 23–27), the increased risk of infertility in men with CAH. In the presence of T-ART, semen analysis showed a condition of oligoasthenoteratozoospermia or azoospermia in about 85% of the cases. The alterations may be caused by ACTH interference because of a feedback loop and a consequent suppression of gonadotropins and testicular testosterone production, or a compression on the rete testis in the presence of a testicular mass, as described previously (3, 24). In the present study, the mechanism seems to be mostly due to a compression, since the gonadotropins were within the normal range, both in basal condition and after 6 months.

The main limitation of this study is the low sample number; this is due to the low incidence of CAH, and it could be difficult to recruit patients for a study when the disease has low incidence. Moreover, not all patients accepted to perform the follow-up for semen analysis; therefore, we cannot conclude that the reduction/disappearance of T-ART was correlated with a significant improvement of sperm parameters. Further studies with more T-ART patients are needed to clarify this aspect.

In conclusion, this study confirms the high prevalence of T-ART in patients with CAH and an association with impaired semen parameters. However, these lesions are potentially reversible, as demonstrated by the disappearance/reduction post-adjustment of cortisone therapy and by the reduction in plasma ACTH level. Therefore, ACTH level should be maintained, as much as possible, within the normal range, as opposed to the usual suggestion in the treatment of CAH patients without T-ART. Finally, the study supports the importance of periodic US evaluation and maintenance of plasma ACTH levels within the normal range in men with CAH, as suggested in the guidelines (2, 28). The differential diagnosis between T-ART and other testicular sonographic anomalies is crucial to avoid unnecessary surgery.

## Data Availability

The datasets generated for this study are available on request to the corresponding author.

## Author Contributions

FM, VT, and RM conceived the study. RM and AS drafted the manuscript. All authors contributed to data collection and/or interpretation and provided a critical revision of the manuscript.

### Conflict of Interest Statement

The authors declare that the research was conducted in the absence of any commercial or financial relationships that could be construed as a potential conflict of interest.
